# Supplementary material for: Spin transport in a lateral spin valve with a suspended Cu channel
Source: Sci Rep. 2020 Jul 1;10:10699. doi: 10.1038/s41598-020-67762-4 (PMC7330037; doi:10.1038/s41598-020-67762-4)
Supplement: Supplementary file 1 — Supplementary information. [file 41598_2020_67762_MOESM1_ESM.pdf]

Supplemental Information

Spin transport in a lateral spin valve with a suspended Cu channel

Kenjiro Matsuki,[1†] Ryo Ohshima,[1†] Livio Leiva,[1] Yuichiro Ando,[1]

Teruya Shinjo,[1] Toshiyuki Tsuchiya,[2] and Masashi Shiraishi[1*]

1. Department of Electronic Science and Engineering, Kyoto Univ., 615-8510 Kyoto, Japan.
2. Department of Micro Engineering, Kyoto Univ., 615-8540 Kyoto, Japan,

[†]Authors contributed equally

[*]Correspondence to shiraishi.masashi.4w@kyoto-u.ac.jp

1. Control experiment (minor loop measurement)

We carried out a minor loop (ML) measurement[1] to confirm that the observed magnetoresistance is attributed to the spin transport through the suspended Cu channel (see Fig. S1). The ML measurement was carried out using the same set-up with the non-local 4-terminal measurement (NL4T). The difference between the NL4T and the ML measurement is a range of the magnetic field: magnetization of one ferromagnetic electrode is fixed in the ML measurement. Here, the negative magnetic field is enough to switch magnetizations of two Py electrodes, but the positive one is not enough to switch the magnetization of the Py electrode with higher switching field. Then, the magnetization of one Py electrode is fixed, and the other one is switched at certain magnetic field, resulting in the hysteresis loop due to the different magnetoresistances in parallel and anti-parallel magnetization configurations. We observed the hysteresis loop in the magnetoresistance and its amplitude is the same as the magnetoresistance in NL4T. In the ML measurement, we can neglect the anisotropic magnetoresistance at a switching magnetic field of the Py electrodes, because the magnetization of the ferromagnetic electrode is fixed. A small difference of the switching magnetic fields (~20 Oe) between the NL4T and ML measurements is attributable to the deviation of the actual magnetic field from the setting value, that is given by the current injected into the electromagnet. Then, since the starting value of the magnetic field was different in the two measurements, the different switching fields can be produced by a different remanence due to the electromagnet hysteresis. Since we carried out this ML measurement half a year after measuring the NL4T shown in the main text, the amplitude of the magnetoresistance was changed (Fix-LSV) and one of the devices was disconnected (Sus-LSV). So, we measured another device fabricated at the same

time and same design with the Sus-LSV discussed in the main text. This is the reason why we prepare the Supplemental Information for the ML and do not include it in the main text. As can be seen, the switching field and the amplitudes of the spin signal in both measurements are the same, and we are convincing that the observed NL4T spin signals is certainly ascribed to the successful spin transport.

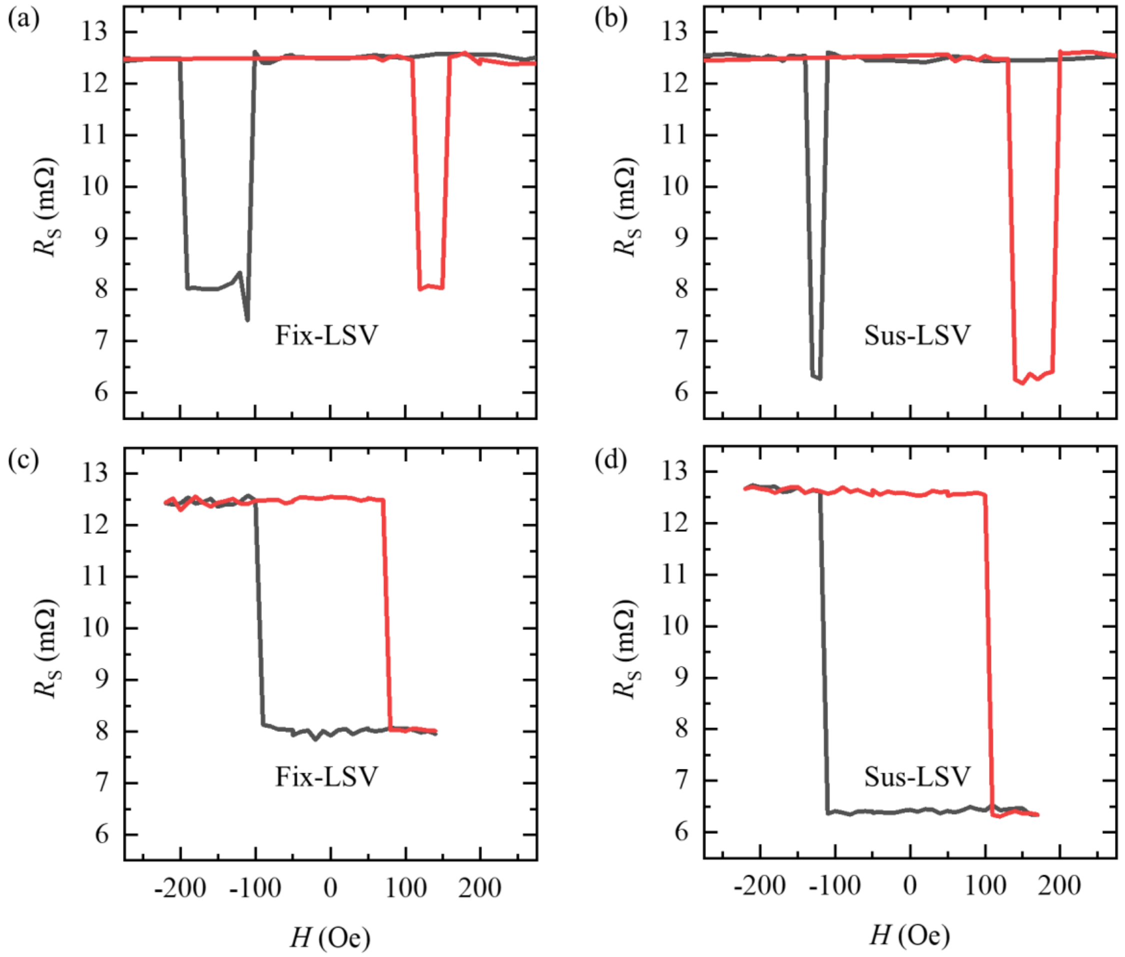

Figure S1: Non-local 4-terminal measurement in lateral spin valves with (a) a fixed Cu channel (Fix-LSV) and (b) a suspended Cu channel (Sus-LSV). The sample structure and measurement setup are same as shown in the main text. A gap length of Py electrodes is designed to be 300 nm. Minor loop signals are obtained by changing the sweep range of the magnetic field from (c) the Fix-LSV and (d) the Sus-LSV.

Another control experiment is expected to be the Hanle spin-precession measurement, however, it is not easy to demonstrate the Hanle-type spin precession in a metallic channel due to a short spin lifetime in metallic materials. If we could fabricate a suspended Cu spin valve with a very long Cu spin channel, the Hanle signals may be detected because a necessary

magnetic field is approximately inversely proportional to a spin channel length if spin diffusion length is not much shorter than the channel length. However, it is not easy to fabricate such a spin valve since (1) the spin diffusion length is 340 nm and (2) such a long Cu channel may not be suspended any more.

2. SEM image of a Cu-based lateral spin valve

To obtain the difference of the switching field of two Py electrodes to observe the rectangular magnetoresistance, we designed the Py electrodes with different shape anisotropy as follows: one is winding and the other is connected to squares (see Fig. S2).

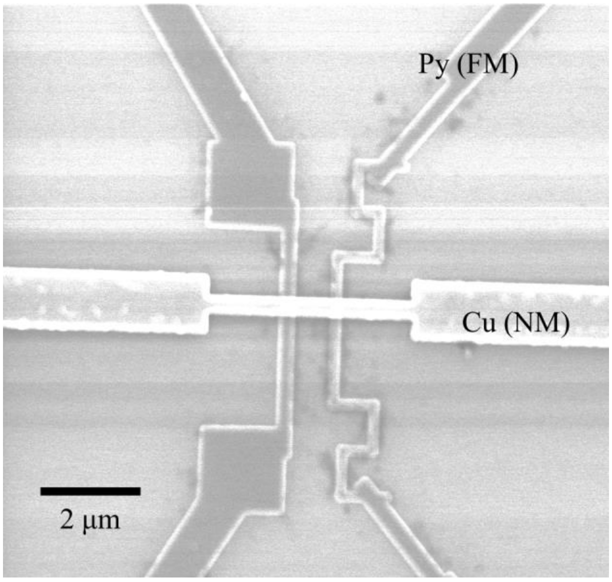

Figure S2: SEM image of a Cu-based lateral spin valve. One of the Py electrodes is winding and the other is connected to squares to change the shape anisotropy.

Reference

1. Sasaki, T., Ando, Y., Kameno, M., Tahara, T., Koike, H., Oikawa, T., Suzuki, T., & Shiraishi, M. Spin Transport in Nondegenerate Si with a Spin MOSFET Structure at Room Temperature. Phys. Rev. Applied **2**, 034005 (2014).
